# Supplementary material for: pH drives electron density fluctuations that enhance electric field-induced liquid flow
Source: Nat Commun. 2024 Jul 15;15:5951. doi: 10.1038/s41467-024-50030-8 (PMC11251051; doi:10.1038/s41467-024-50030-8)
Supplement: Supplementary file 1 — Supplementary Information [file 41467_2024_50030_MOESM1_ESM.pdf]

Supplementary Information for

pH drives electron density fluctuations that enhance electric field-induced liquid flow

S. Pullanchery[1*], S. Kulik[1*], T. Schönfeldová[1], C. K. Egan[4], G. Cassone[5], A. Hassanali[4#], S. Roke[1,2,3#]

This PDF file includes:

Supplementary Notes 1-5

Supplementary Figures 1-6

Supplementary Tables 1-6

Supplementary References

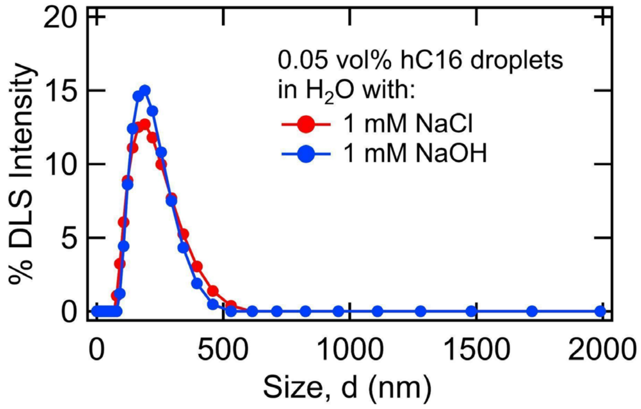

Supplementary Figure 1. Oil droplet size distribution. DLS intensity distribution as a function of diameter of 0.05 vol % hexadecane droplets prepared in water with 1 mM NaCl (red curve) and 1 mM NaOH (blue curve). The PDI of the curves correspond to 0.2 (NaCl) and 0.2 (NaOH), respectively.

Supplementary Note 1: Computing $S(\theta)$ curves

The computed $S(\theta)$ curves (black lines) in Fig.1F were obtained using nonlinear light scattering theory, described in detail in Refs[1-2]. The equations used to relate $S(\theta)$ to the surface potential are derived using the Rayleigh-Gans-Debye (RGD) approximation, in the absence of multiple scattering, with a first-order correction for the jump in the electromagnetic field amplitude across the interface[3]. Equations S3 and S4 are derived by considering the following assumptions[1-2,4]:

- The liquid can be considered spatially isotropic in the azimuthal direction. This ensures that only 4 of the possible 81 elements of the $\chi^{(3)\prime}$ and $\Gamma^{(3)\prime}$ tensors are non-zero.
- The particle interface can be considered isotropic in the interfacial plane. This reduces the complexity such that only 4 elements of the $\chi_s^{(2)}$ and $\Gamma^{(2)}$ are non-zero.
- The sample consists of a lossless nonlinear medium, which means that no energy is transferred from the optical pulses to the material. This leads to the degeneracy of three of the four tensor elements of $\chi_s^{(2)}$ ($\Gamma^{(2)}$), and $\chi^{(3)\prime}(\Gamma^{(3)\prime})$, so that only the pair ($\chi_1^{(2)}$, $\chi_2^{(2)}$), and $\chi_2^{(3)\prime}$ are left as non-zero.
- The orientation distribution of water molecules at the interface is broad. Under these conditions, the tensor element $\chi_{s,1}^{(2)}$ becomes negligible.

The equations that relate $S(\theta)$ to R, Φ_0 and $\chi_{s,2}^{(2)}$ are given in Ref.[2] (Equations 2 and 3, and Tables 2 and 3). This formalism was later modified for water-in-oil droplets, where contributions from both the inside aqueous and outside oil interfaces were considered[5]. Here, we adapt a similar approach to calculate scattering patterns that take into account both the oil and water contributions from the oil/water interface. In this formalism, the scattered intensity for the two independent polarization combinations PPP and PSS can be expressed as:

$$S_{PPP}(\theta) = \frac{I_{PPP}(\theta)}{I_{SSS}(\theta)} = \frac{\varepsilon_0^2\left(E_P(\omega)^2\left[cos\left(\frac{\theta}{2}\right)^3\left(\Gamma_{1,oil}^{(2)}-\Gamma_{1,H_2O}^{(2)}\right) + cos\left(\frac{\theta}{2}\right)\left(\left(\Gamma_{2,oil}^{(2)}-\Gamma_{2,H_2O}^{(2)}\right) + \left(\Gamma_{2,H_2O}^{(3)\prime}+\Gamma_{2,oil}^{(3)\prime}\right)\right)(2cos(\theta)+1)\right]\right)^2}{\bar{\mu}^2 N_b/N_p} \quad \text{(S1)}$$

$$S_{PSS}(\theta) = \frac{I_{PSS}(\theta)}{I_{SSS}(\theta)} = \frac{\varepsilon_0^2\left(E_S(\omega)^2\left[cos\left(\frac{\theta}{2}\right)\left(\left(\Gamma_{2,oil}^{(2)}-\Gamma_{2,H_2O}^{(2)}\right)+\left(\Gamma_{2,H_2O}^{(3)\prime}+\Gamma_{2,oil}^{(3)\prime}\right)\right)\right]\right)^2}{\bar{\mu}^2 N_b/N_p} \quad \text{(S2)}$$

where $\Gamma_{1,H_2O}^{(2)}$, $\Gamma_{2,H_2O}^{(2)}$, and $\Gamma_{2,H_2O}^{(3)\prime}$ are the effective particle susceptibilities of water, and $\Gamma_{1,oil}^{(2)}$, $\Gamma_{2,oil}^{(2)}$, and $\Gamma_{2,oil}^{(3)\prime}$ are the effective particle susceptibilities of the oil inside the droplet. $\bar{\mu} = \bar{\beta}_{H_2O}^{(2)} E(\omega)^2$ is the average induced second-order dipole moment with $\bar{\beta}_{H_2O}^{(2)}$ the averaged hyperpolarizability of water. N_p is the number of droplets and N_b is the density of bulk water (3.34 x 10^{28} molecules/m^3). The elements of the nonlinear effective particle susceptibility are related to the surface susceptibility according to the expressions in table S1.

Supplementary Table 1: Analytical expressions used for computing equations S3 and S4 comprised of the surface and particle susceptibility elements of the interface, the form factor functions and the scattering vector.

**Susceptibility elements outside the droplet**	**Susceptibility elements inside the droplet**
$\chi_{s,1}^{(2)''} = 27\eta\frac{\left(\chi_{s,1}^{(2)}\eta^2 + 3\chi_{s,2}^{(2)}(\eta^2-1)\right)}{(2+\eta)^3}, \eta = \left(\frac{n_p}{n_{H_2O}}\right)^2$	$\Gamma_1^{(2)} = 0$
$\chi_{s,2}^{(2)''} = 27\eta\frac{\chi_{s,2}^{(2)}}{(2+\eta)^3}$	$\Gamma_2^{(2)} = 0$
$\chi_2^{(3)''} = 27\eta\frac{\chi_2^{(3)\prime}}{(2+\eta)^3}, \chi_2^{(3)\prime} = \frac{N_b}{\varepsilon_0}\left(\bar{\beta}^{(3)} + \frac{\bar{\beta}^{(2)}\mu_{dc}}{3k_BT}\right)$	$\Gamma_2^{(3)\prime} = 2\chi_2^{(3)''}\Phi_0(F_1(qR))$
$\Gamma_1^{(2)} = (2F_1(qR) - 5F_2(qR))\chi_{s,1}^{(2)''}$	$\chi_2^{(3)''} = 27\eta\frac{\chi_2^{(3)\prime}}{(2+\eta)^3}, \chi_2^{(3)\prime} = \frac{N_b}{\varepsilon_0}(\bar{\beta}^{(3)})$
$\Gamma_2^{(2)} = F_2(qR)\chi_{s,1}^{(2)''} + 2F_1(qR)\chi_{s,2}^{(2)''}$	
$\Gamma_2^{(3)\prime} = 2\chi_2^{(3)''}\Phi_0(F_1(qR) + F_3(qR,\kappa R))$	

Supplementary Table 2: Analytical expressions of the form factor functions and the scattering vector used for computing equations S3 and S4.

**Form factor functions and scattering vector**
$F_1(qR) = 2\pi R^2 i\left(\frac{sin(qR)}{(qR)^2} - \frac{cos(qR)}{qR}\right)$
$F_2(qR) = 4\pi R^2 i\left(3\frac{sin(qR)}{(qR)^4} - 3\frac{cos(qR)}{(qR)^3} - \frac{sin(qR)}{(qR)^2}\right)$

$F_3(qR, \kappa R) = 2\pi R^2 i \dfrac{qRcos(qR) + \kappa Rsin(qR)}{(qR)^2 + (\kappa R)^2}$
$q = k_0 - 2k_1,\ q = \left\lvert\frac{4\pi\eta_{H_2O}}{\lambda_{SH}} sin\frac{\theta}{2}\right\rvert$

The parameters used to compute the SHS scattering patterns of oil droplets in water (Fig. 1F) are given in supplementary table 4.

Supplementary Table 3: Constants and equalities used in equations S3 and S4 (*16*)

**Constants and equalities**
$\mu_{dc} = 8.97 \times 10^{-30}\ Cm$
$\bar{\beta}^{(2)} = 3.09 \times 10^{-52}\ C^3m^3J^{-2}$ for H_2O
$\bar{\beta}^{(3)} = 4.86 \times 10^{-62}\ C^4m^4J^{-3}$ for H_2O<br>$\bar{\beta}^{(3)} = 7.09 \times 10^{-61}\ C^4m^4J^{-3}$ for n-$C_{16}H_{34}$ [10]
$\chi^{(2)}_{s,1} = \chi^{(2)}_{s,\perp\perp\perp} - \chi^{(2)}_{s,\parallel\parallel\perp} - \chi^{(2)}_{s,\parallel\perp\parallel} - \chi^{(2)}_{s,\perp\parallel\parallel}$
$\chi^{(2)}_{s,2} = \chi^{(2)}_{s,\parallel\parallel\perp}$
$\chi^{(2)}_{s,1} = 0$
$\chi^{(2)}_{s,4} = \chi^{(2)}_{s,3} = \chi^{(2)}_{s,2},\ \chi^{(3)\prime}_{4} = \chi^{(3)\prime}_{3} = \chi^{(3)\prime}_{2}$

Supplementary Table 4: Parameters used to compute the SHS curves of oil droplets in water

**Parameter**	**Value**
SH wavelength, λ_{SH}, nm	514
Refractive index of water, n_{H_2O}	1.333
Refractive index of oil, n_{oil}	1.435
Radius of droplets, R, nm	DLS distribution
Number density of droplets, N_p, mL^{-1}	$1.7 \cdot 10^{10}$
Ionic strength of water, mM	1
Ionic strength of oil, mM	0
Surface susceptibility of water, $\chi^{(2)}_{s,2,H_2O}$, m^2/V	$-0.24 \cdot 10^{-22}$
Surface susceptibility of oil, $\chi^{(2)}_{s,2,oil}$, m^2/V	0

Supplementary Note 2: Retrieving the interfacial water spectrum of oil droplets

Broadband IR pulses between 2200 and 3000 cm^{-1} spaced with 100 cm^{-1} frequency steps were used to probe the entire O-D stretching region. The infrared spectral profiles were recorded as the sum frequency intensity from a solid film of $BaTiO_3$ nanoparticles and are shown in Supplementary Fig.2A. The measured sum frequency spectrum at each frequency range was background subtracted and then normalized with IR and VIS pulse energy and acquisition time. The total SF intensity at each pulse center frequency ω_0 was computed as a weighted sum:

$$I_{SF(\omega=\omega_0)} = \frac{\sum_{i=1}^{n} I_{SF,i(\omega_0)}\, I_{0(\omega_{IR}),i}}{\sum_{i=1}^{n} I_{0(\omega_{IR}),i}} \quad \text{(S3)},$$

where the index i runs through all IR excitations that contribute to the intensity at $\omega = \omega_0$ and $I_{0(\omega_{IR}),i}$ is the incident IR intensity that generates sum frequency signal at $\omega = \omega_0$. The resultant spectrum after the summation is shown in Supplementary Fig.2B.

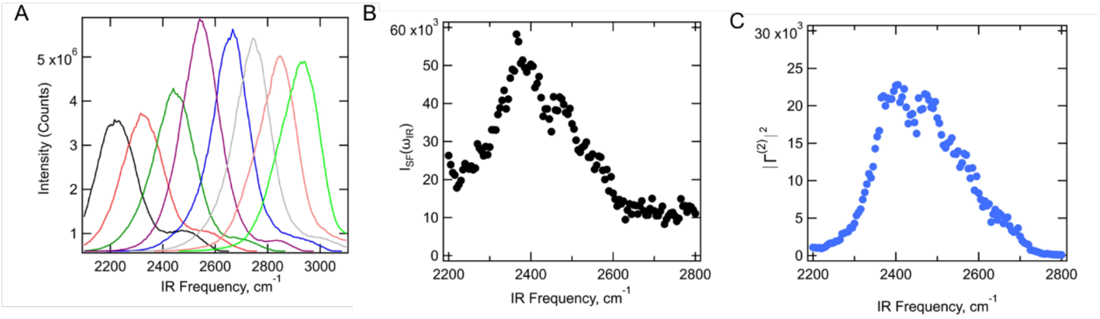

Supplementary Figure 2: **Measured IR and SF spectra, and converted SFS water spectrum**. Procedure for retrieving the interfacial water spectrum of 2% hexadecane droplets in D_2O. A) The SFS signal from $BaTiO_3$ nanoparticle film, representing the shape of IR pulses used for SFS measurements B) The total summed SFS spectrum (Eq. S3) for the entire O-D stretch region. C) The resultant $|\Gamma^{(2)}|^2$ SF spectrum of hexadecane oil droplets in 1mM NaOD in D_2O retrieved by dividing the SF measurement (B) by the function $C(\omega_{IR})$ (Eq., S4, Supplementary Fig.3C).

Converting the measured SF intensity to interfacial $|\Gamma^{(2)}|^2$ spectra.

The relation between SF intensity and the true nonlinear interfacial scattering response is given by the following equation:

$$\left|\Gamma^{(2)}(\omega_{SF};\omega_{VIS},\omega_{IR})\right|^2 \propto \frac{I_{SF}(\omega_{SF};\omega_{VIS},\omega_{IR})}{\int_0^L I_{IR}(\omega_{IR},z) I_{VIS}(\omega_{VIS}) f_{focal}(z)\rho(z)\,dz} = \frac{I_{SF}(,\omega_{IR})}{C(\omega_{IR})} \quad \text{(S4)}$$

where $\omega_{SF}, \omega_{VIS}$, and ω_{IR} are the frequencies of the sum frequency, visible, and infrared pulses. $I_{SF}(\omega_{SF}; \omega_{VIS}, \omega_{IR}) = I_{SF}(\omega_{IR})$ is the measured SF intensity. $I_{IR}(\omega_{IR}, z)$ is the intensity of the incident infrared light along the optical z axis which is given by Lambert-Beers' law, $I_{IR}(\omega_{IR}, z) = I_0(\omega_{IR})e^{-\alpha(\omega_{IR})z}$, with, $I_0(\omega_{IR})$ being the incident IR intensity from the laser and $\alpha(\omega_{IR})$ the absorption spectrum. $\alpha(\omega_{IR})$ is computed from the measured IR transmission spectrum (T, Supplementary Fig. 3) as $\alpha(\omega_{IR}) = \frac{-ln\,(T(\omega_{IR}))}{L}$. $I_{VIS}(\omega_{VIS})$ is the intensity of the incident visible light, $f_{focal}(z)$ is a measured collection function that describes the efficiency of light collection along the optical z axis of the collection optics (Supplementary Fig. 3), and $\rho(z)$ is the number density of particles at a specific depth, which is assumed to be uniform (i.e. $\rho(z) = 1$). $C(\omega_{IR})$ is the final correction factor resulting from numerically integrating the denominator in the middle of Eq. S4 (Supplementary Fig. 3). $\boldsymbol{\Gamma}^{(2)}(\omega_{SF}; \omega_{VIS}, \omega_{IR}) = \boldsymbol{\Gamma}^{(2)}$ is the effective second-order particle susceptibility that describes the spectral interfacial response of droplets dispersed in solution. $\boldsymbol{\Gamma}^{(2)}$is a function of the scattering angle (θ, defined as the angle between the scattered SF wavevector and that of the phase-matched direction, Fig. 1B), the size of the droplets (typically indicated by R, the average droplet radius), and the second-order surface susceptibility ($\boldsymbol{\chi}^{(2)}$) [6-8] .

Eq. S4 contains a number of parameters that where experimentally determined. The absorption coefficient $\alpha(\omega_{IR})$, was computed from transmission spectra of water. The transmission spectrum was recorded using a Bruker Vertex 70 FTIR spectrometer, with a transmission sample cell that has CaF_2 (front) and quartz (back) windows with an optical path length of 10 microns. The windows are the same windows as used for the SFS sample cell. The spectrum is shown in Supplementary Fig. 3A. Another parameter is $f_{focal}(z)$, the collection function that describes the efficiency of light collection along the optical axis z of the collection optics. This function was measured using a sample of stearyl covered silica particles (R=123 nm) dispersed in CCl_4 [9]. By recording the SFS spectrum while translating the sample along the optical axis (see also Ref. [10]), $f_{focal}(z)$, was determined. Supplementary Fig. 3B shows the integrated spectral intensities and the fit function that was used as $f_{focal}(z)$. Supplementary Fig. 3C shows the computed correction factor $C(\omega_{IR})$ from eq.S4. The measured SF intensity and the corrected $|\boldsymbol{\Gamma}^{(2)}|^2$ spectra are shown in Supplementary Fig. 2B and 2C respectively.

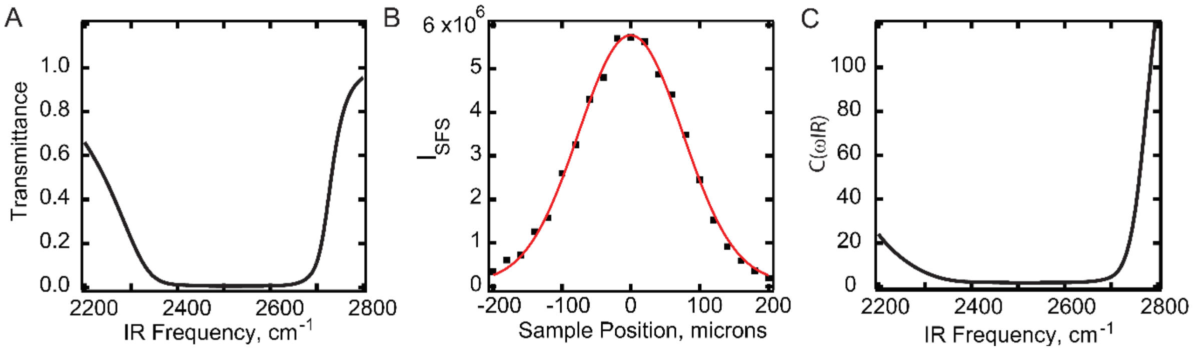

Supplementary Figure 3: Parameters for Eq. S4. A) FTIR transmittance profile of D_2O measured using sample cell similar to the one used for SFS measurements but with a 10 μm path length. B) the integrated spectral intensities and the fit function that was used as $f_{focal}(z)$. C) $C(\omega_{IR})$ as computed from eq. S4.

Supplementary Note 3. Examples of the surface chemical sensitivity of SFS

To demonstrate the surface sensitivity of vibrational SFS on the interfacial structure, we show in Supplementary Fig. 4 SFS spectra of 100 nm d34-hexadecane droplets in aqueous (D_2O) solution. Supplementary Fig.4 A and 4B show the same droplets, but in contact with 1 mM solutions of zwitterionic phospholipids (1,2-dipalmitoyl-sn-glycero-3-phosphocholine (DPPC) and 1,2-dipalmitoyl-sn-glycero-3-phosphoethanolamine (DPPE), Supplementary Fig.4A), as well as charged phospholipids (1,2-dipalmitoyl-sn-glycero-3-phosphate (DPPA), 1,2-dipalmitoyl-sn-glycero-3-phospho-(1'-rac-glycerol) (DPPG), and 1,2-dipalmitoyl-sn-glycero-3-phospho-L-serine (DPPS), Supplementary Fig.4B), and a surfactant sodium dodecyl sulfate (SDS). Both panels show very different spectra that report on the different surface structures of the different lipid acyl chains[11].

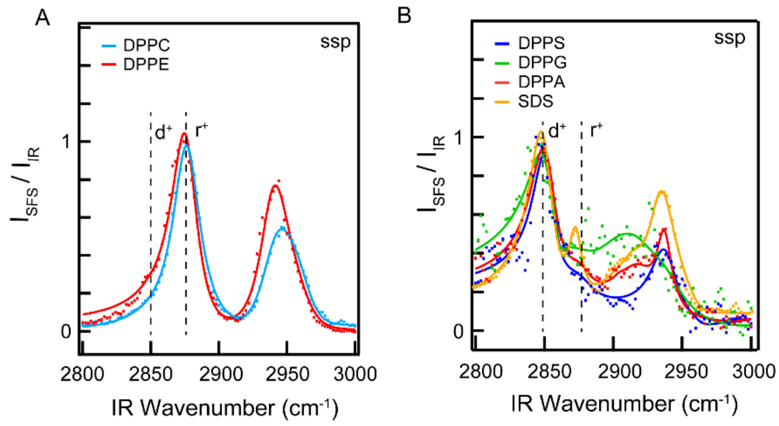

Supplementary Figure 4. The exceptional sensitivity of SFS to surface chemistry. A) Normalized SFS spectra in the C−H stretching region of 1 mM DPPC and DPPE monolayers on 100 nm radius d34-hexadecane nanodroplets (2 vol %) in D_2O. The spectra are normalized to the s-CH_3 stretching mode (r^+). (B) Normalized SFS spectra of DPPS (1 mM), DPPG (1 mM), DPPA (1 mM), and SDS (10 mM) monolayers on oil (d_{34}-hexadecane)

nanodroplets (2 vol %) in D_2O of the C−H stretching modes. The spectra are normalized to the s-CH_2 stretching mode (d^+). (A) and (B) are adapted from Ref.[11].

Supplementary Note 4. Relation to previous experiments and considerations around impurities

Historically, the pH-dependent increase in droplet mobility has been explained in terms of an increase in charge density. Because the droplet speed in an electrostatic field depends linearly on the adsorbed surface charge, this would explain the mobility increase. An increase in charge density necessarily also results in an increase in the surface potential. For low surface charge densities and at constant ionic strength, $\sigma_0 \sim \Phi_0$. Therefore, if σ_0(pH 11) = 2.2 x σ_0(pH 7), then Φ_0 (pH 11) = 2.2 x Φ_0 (pH 7). In Fig. 2, we directly measure Φ_0, and this measurement does not require any input on how Φ_0 relates to σ_0. The measured intensity scales with Φ_0^2, and thus, we expect at least an increase in the intensity by a factor of ~4, which is indeed what the data in Fig. 1D (green data, Φ_0 = -60 mV; blue data Φ_0 = -109 mV) and the model of Fig. 1F (dashed/solid lines) provide. Therefore, while the increase in droplet mobility agrees with literature, and could in the absence of any other experiments be explained by a pH-dependent increase in σ_0, it is not reconcilable with the AR-SHS measurements of Fig. 1F. In addition, the SFS measurements are also sensitive to Φ_0, in a very similar manner as the AR-SHS measurements, and an increase in Φ_0, would have been clearly visible as a change in the O-D stretch intensity. In fact, this is seen in Fig. 2B, where the SFS O-D spectrum of 1 vol% 200 nm radius hexadecane droplets in a 1 mM AOT-water solution is plotted. Here, the O-D response has increased drastically compared to that of neat ultrapure oil droplets in water. Similar vibrational SFS experiments have been conducted in the past decades in Refs[12-13]. Various pH-dependent aspects where reported, always in combination with ζ-potential measurements: The phase between oil and water responses[13], and the weakly resonant D-O tail of the vibrational SF response[12]. Both these metrics should be changing if the surface charge changes. While the ζ-potential was pH dependent and reproduced literature values, neither the oil-water phase[13] nor the weakly resonant SF D-O response showed any sign of pH dependence in the pH range 7-12[12], which is consistent with the present observation and explanation.

Next, we briefly comment on the aspect of impurities. ζ-potentials as measured at different pH values are in the range |30 -120| mV[14]. Applying the Poisson-Boltzmann equation[15], this corresponds to surface charge densities in the range ~ 0.01 (100) – 0.3 (3.33) e^-/nm^2 (nm^2/e^-). Since these are low values, several studies, some theoretical[16-17], have brought forth the possibility that impurities are responsible for the pH dependence in the ζ-potential. These impurities are hypothesized to arise from the oil (in the form of carboxylates[18]),

the water (in the form of an undefined agent with a specific pK_a[16], or atmospheric CO_2[19], or from what appears to be the walls of the glass cuvette when it is subjected to > 24h of sonication[20-21]. In neither of these studies independent surface potential measurements where conducted, and direct molecular level evidence was absent.

Impurities are unwanted substances that derive from local geographic conditions and are indeed an inescapable element in any experiment with chemicals. In most scientific domains experimental reproducibility of data is taken as evidence for the relative unimportance of impurities, since they tend to vary in time and space. Here, the pH-dependent change in electrophoretic effects is seen in diverse experiments[14,22-31], conducted across different eras (1930's – 2020's), and on different continents (Europe, Oceania, America, Asia). Thus, it would be highly unlikely if impurities are at the origin of the increase in mobility or conductivity.

Role of impurities in reflection SFG experiments on planar oil-water interfaces vs SFS from oil nanodroplets

Experiments that do show a lot of variability are reflection SFG experiments[32-37] conducted on planar oil-water interfaces, as discussed in detail in Ref.[38] (section S1). Various groups have aimed to measure the water / oil molecular structure and reported vastly different SFG spectra, even a few years apart within the same laboratory (plotted in Fig. S1 of Ref.[38], data in plots from Refs.[32-33,35-37]). Due to the difficulty of making a pristine planar oil water interface in combination with the very small surface to volume ratio, we expect that in this case, impurities might play a role, in causing the different spectral shapes. Note that the surface area to be considered here is the total area of oil-water interface, and the volume to be considered is the total volume of the liquid used to make that interface. In contrast to extended planar oil-water interfaces, oil nanodroplets in water are easy to prepare reproducibly, have a ~ 10^4 x higher surface to volume ratio than planar interfaces and allow for measuring electrophoresis, electrostatic potential and molecular structure on the same sample. Based on the above discussion (reproducible data, high surface to volume ratio) we expect the influence of impurities to be unimportant for nanodroplets, but important for planar extended interfaces. Nevertheless, we have in the past published several studies in which we quantified the role of impurities in our experiments (see Refs.[21,39-41]): We examined the effect of impure oil[39], the possibility of impure water and salt, the effect of dissolved CO_2[40], as well as impurities leaching from glass wall containers[21]. We also measured the level of impurity in the chemicals used in our experiments, and determined the sensitivity of our experimental tools: The concentration of impurities in our samples is ~< 1 nM, primarily a consequence of the sensitivity of the methods to determine these[40]. Furthermore, we determined the surface sensitivity of our techniques on 100 nm radius objects in a 1 vol % dispersion (SFS), or a 0.05 vol % dispersion (SHS). We previously[39] determined that vibrational sum frequency scattering has a sensitivity

of detecting 1 molecule / 27 nm^2, which is presently improved by a factor of ~5 by varying detection settings[42]. For AR-SHS the sensitivity is better: AR-SHS can detect a single molecule adsorbing on a ~ 100 nm object, i.e. 1 molecule / 10^5 nm^2 (in the femtomolar range in terms of bulk concentration[43]. Equivalent non-resonant SH imaging detects a single oriented water molecule in a ~320 nm^3 bulk volume[44], which converts to a similar sensitivity value for a droplet interface. Therefore, we conclude that on the basis of reproducibility of results (in our lab over a period of almost 20 years), samples with surface to volume ratios ~ ~10^4 x higher than in other surface sensitive measurements, and the excellent (SFS) and exquisite interfacial sensitivity (SHS) the role of impurities in this context is negligible.

Reflection SFG studies on the planar extended hexane-water interface as a function of pH

Two recent studies have performed femtosecond reflection mode SFG experiments on the hexane/water interface, whereby the hexane was dosed from the gas phase to form films with sub monolayer to monolayer coverages[36-37]. It was shown by both Ref.[36] and Ref.[37] that the SFG intensity at the hydrophobic liquid/water interface is stronger than the air/water interface due to the water ordering by hydrophobic groups. The 2020 study by Yang et al.[37] additionally showed that the SFG spectrum of water at a hexane/water interface increases in intensity (but not in shape) with increasing pH at constant ionic strength. Although there was no direct surface potential and streaming potential measurement, the increase in intensity was attributed to the interfacial presence of ionic charge carriers, and modelled using a Gouy-Chapman model to extract surface charge densities. It was also assumed that the surface susceptibility was pH-independent (meaning that $\chi_s^{(2)}$ is constant). Interestingly, Ref.[37] also showed that hexane chains drastically reorient as a function of pH. At pH 5.6, the alkyl chains are nearly perpendicular to the interface, and they become more parallel to the interface at pH 10. Such a reorientation has two consequences: it likely changes the value of $\chi_s^{(2)}$ as a function of pH, and it can significantly alter the interfacial water ordering. The SFG data was further analysed to obtain an adsorption free energy, which had the value of 37 kJ/mol (~ 14 kT), which should lead to a surface density of ~ 2 OH^- ions / nm^2. With such densities, considering the sensitivity of SFG, one might expect a spectral signature of interfacial OH^-. SFG reports on the product of IR and Raman cross-sections. OH^- has a vibrational mode at 3645 cm^{-1}, which is Raman active (narrow peak on the blue spectral side with a strong absorption cross section) and IR active (shoulder on the blue spectral side having a weaker absorption cross section than in Raman spectroscopy), yet it hasn't been detected in the SF spectra of hydrophobic/water interfaces. IR and Raman spectroscopy in crystals of $Ca(OH)_2$, $Mg(OH)_2$ show the hydroxide O-H stretch mode. SFG from the CaF_2/water interface immersed in basic

aqueous solution has also shown a hydroxide feature[45-47]. Although this could be either surface adsorbed hydroxide or hydroxide within the surface lattice, it suggests that surface hydroxide species are SFG active and detectable when present. Therefore, it is curious that a molecular group that is SFG active with a surface adsorption free energy of 14kT does not produce a sum frequency mode corresponding to the adsorbed OH^- anion at hydrophobic/water interfaces.

On the other hand, the absence of a hydroxide peak might not provide conclusive evidence for the absence of OH^- at the surface. The lack of spectral signature can also be due to other causes, such as the lack of a specific orientation combined with an unexpectedly low cross-section. Therefore, only spectral measurements in combination with independent surface potential measurements or other direct probes of OH^- can conclusively prove the presence/absence of OH^- ions. At the planar hexane/water interface, such independent measurements are yet to be made. Therefore, the increase in the O-H stretch signal at the planar interface can only be viewed as an increased ordering of hydrogen-bonded water at the interface, which can also have its origins in the reorientation of alkyl chains rather than OH^- absorption. In the present work, however, no change in the alkane orientation is observed as a function of pH (Fig. 2D). Both studies are therefore not necessarily in disagreement.

Supplementary Note 5. Many Body Energy Decomposition Analysis

In the main text, we present the average 2-body (2B) E_{pol} and E_{ct} terms as a function of distance between neopentane and water. Here, we show the similar averages for all 2B ALMO EDA terms (including the total 2B interaction energy). These averages were calculated from instantaneous (close-range) neopentane-water dimers from our AIMD simulations. Note that averages for the 0.1V/Å fields correspond to dimer geometries from field-on calculations, while all ALMO EDA calculations were performed in post-processing without any external electric fields. Also note that dimers with a separation around 4 Å are less common than those at larger distances, leading to larger error bars, as shown in the figures.

We estimate the total 3-body (3B) contributions to E_{ct} and E_{pol} using a single configuration taken from the field-on simulation. It was found that 3B E_{ct} corresponds to around -15% of the total E_{ct} energy (a negative percent due to the opposite sign as the total E_{ct}), while 3B E_{pol} corresponds to around +25% of the total E_{pol}. The estimated total 2B and 3B energies are shown in the tables below.

Supplementary Table 5. Estimated total 2B and 3B contributions to E_{pol}

Total 2B E_{pol}	Est. total 3B E_{pol}	Est. total 2B + 3B E_{pol}
-5.9 kJ/mol	-2.0 kJ/mol	-7.9 kJ/mol

Supplementary Table 6. Estimated total 2B and 3B contributions to E_{ct}

Total 2B E_{ct}	Est. total 3B E_{ct}	Est. total 2B + 3B E_{ct}
-7.2 kJ/mol	+1.0 kJ/mol	-6.2 kJ/mol

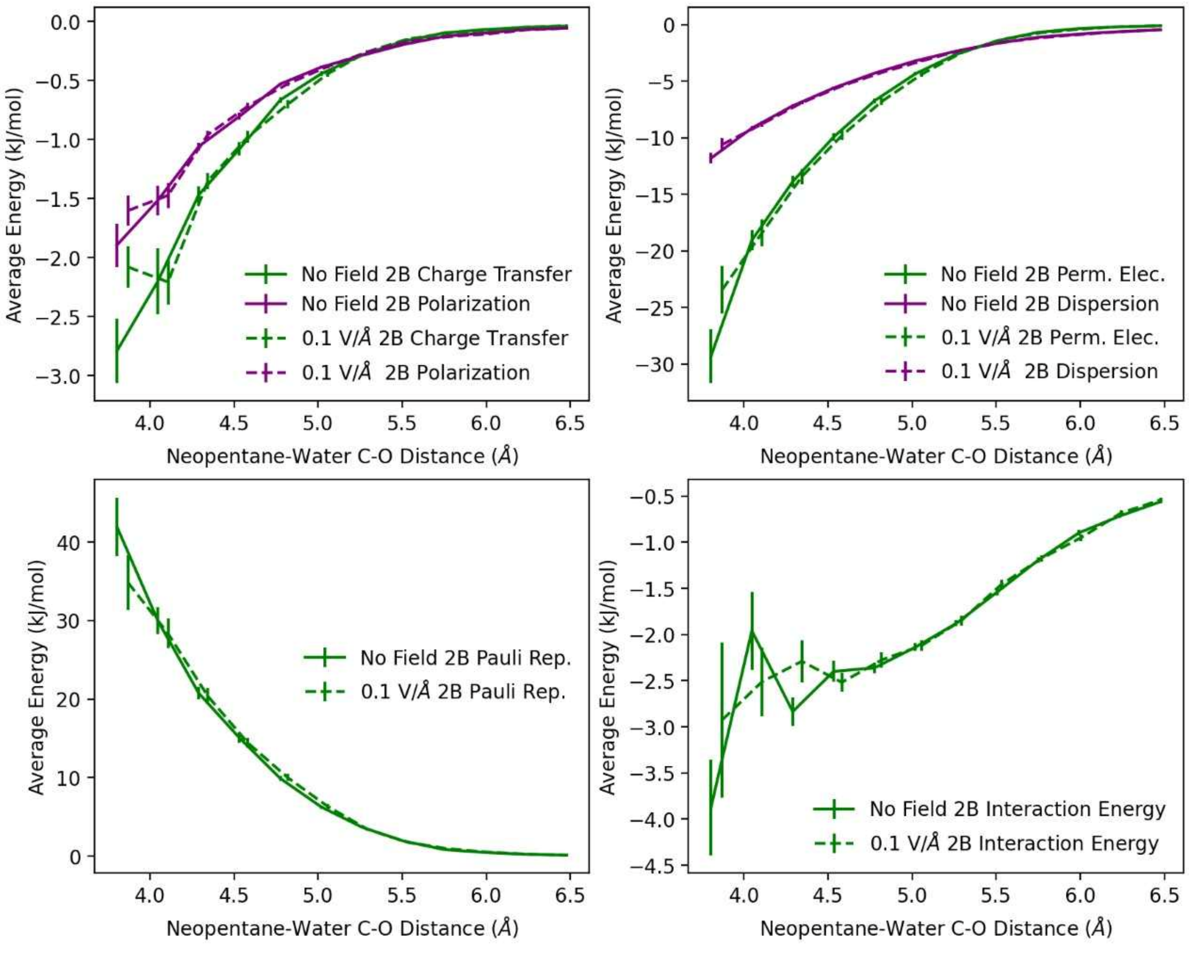

Supplementary Figure 5. Average 2-body (2B) interaction energy per water molecule between the water and neopentane as a function of distance separated into the contributions coming from a) polarization and charge-transfer, b) permanent electrostatics and dispersion, c) Pauli repulsion. The average total 2B interaction energy is shown in panel d. The solid and dashed lines correspond to the average energy contributions of molecular geometries taken from simulations in the absence of an external electric field and in the presence of a 0.1 V/Å field, respectively (note that all polarization and charge transfer energies were calculated without any external field). Also note the difference in scales between the four panels.

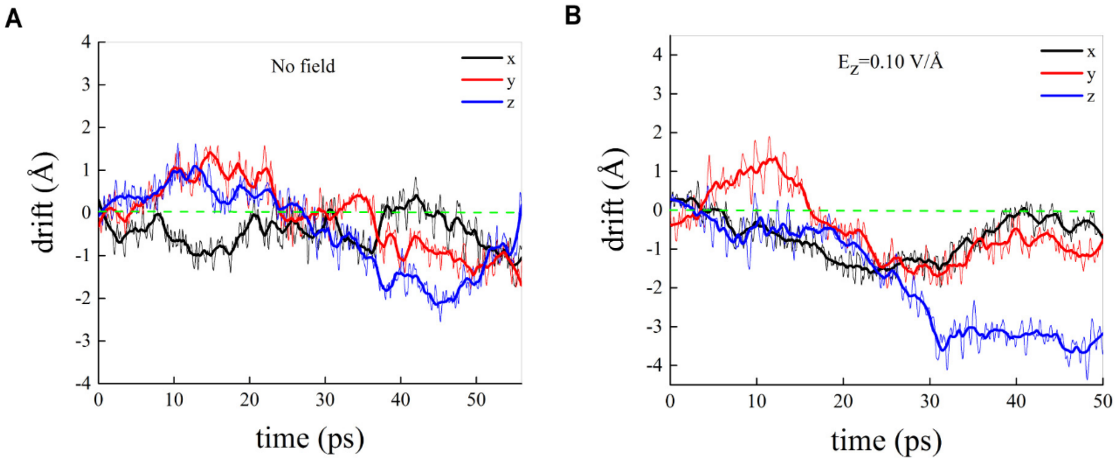

Supplementary Figure 6. Mobility of neopentane. Cartesian components of the drift of the Carbon atoms of the neopentane molecule at zero-field conditions (A) and in presence of an external static and homogeneous electric field of intensity equal to 0.1 V/Å and oriented along the z-axis (B). The net negative drift of the Cartesian component coincident with the field axis (z, blue line) magnifies the negatively charged nature of the neopentane molecule under the field action. A Savitzky-Golay smoothing (thicker lines) of the raw data (thinner lines) is also shown.

Supplementary References

1. Gonella, G.; Lütgebaucks, C.; de Beer, A. G. F.; Roke, S. Second Harmonic and Sum-Frequency Generation from Aqueous Interfaces Is Modulated by Interference. *J. Phys. Chem. C.* **2016,** *120*, 9165-9173.
2. Lütgebaucks, C.; Gonella, G.; Roke, S. Optical Label-Free and Model-Free Probe of the Surface Potential of Nanoscale and Microscopic Objects in Aqueous Solution. *Phys. Rev. B* **2016,** *94*, 195410.
3. Dadap, J. I.; Shan, J.; Heinz, T. F. Theory of Optical Second-Harmonic Generation from a Sphere of Centrosymmetric Material: Small-Particle Limit. *J. Opt. Soc. Am. B* **2004,** *21*, 1328-1347.
4. de Beer, A. G. F.; Campen, R. K.; Roke, S. Separating Surface Structure and Surface Charge with Second-Harmonic and Sum-Frequency Scattering. *Phys. Rev. B* **2010,** *82*, 235431.
5. Smolentsev, N.; Roke, S. Self-Assembly at Water Nanodroplet Interfaces Quantified with Nonlinear Light Scattering. *Langmuir* **2020,** *36*, 9317-9322.
6. Roke, S.; Bonn, M.; Petukhov, A. V. Nonlinear Optical Scattering: The Concept of Effective Susceptibility. *Phys. Rev. B* **2004,** *70*, 115106.
7. de Beer, A. G. F.; Roke, S. Sum Frequency Generation Scattering from the Interface of an Isotropic Particle: Geometrical and Chiral Effects. *Phys. Rev. B* **2007,** *75*, 245438.
8. Dadap, J. I.; de Aguiar, H. B.; Roke, S. Nonlinear Light Scattering from Clusters and Single Particles. *J. Chem. Phys* **2009,** *130*, 214710.
9. Roke, S.; Berg, O.; Buitenhuis, J.; van Blaaderen, A.; Bonn, M. Surface Molecular View of Colloidal Gelation. *Proc. Natl. Acad. Sci. U. S. A.* **2006,** *103*, 13310.
10. Roke, S.; Roeterdink, W. G.; Wijnhoven, J. E. G. J.; Petukhov, A. V.; Kleyn, A. W.; Bonn, M. Vibrational Sum Frequency Scattering from a Submicron Suspension. *Phys. Rev. Lett.* **2003,** *91*, 258302.
11. Chen, Y.; Okur, H. I.; Lütgebaucks, C.; Roke, S. Zwitterionic and Charged Lipids Form Remarkably Different Structures on Nanoscale Oil Droplets in Aqueous Solution. *Langmuir* **2018,** *34*, 1042-1050.

12. Samson, J.-S.; Scheu, R.; Smolentsev, N.; Rick, S. W.; Roke, S. Sum Frequency Spectroscopy of the Hydrophobic Nanodroplet/Water Interface: Absence of Hydroxyl Ion and Dangling Oh Bond Signatures. *Chem. Phys. Lett.* **2014,** *615*, 124-131.
13. Vácha, R.; Rick, S. W.; Jungwirth, P.; de Beer, A. G. F.; de Aguiar, H. B.; Samson, J.-S.; Roke, S. The Orientation and Charge of Water at the Hydrophobic Oil Droplet–Water Interface. *J. Am. Chem. Soc.* **2011,** *133*, 10204-10210.
14. Agmon, N.; Bakker, H. J.; Campen, R. K.; Henchman, R. H.; Pohl, P.; Roke, S.; Thämer, M.; Hassanali, A. Protons and Hydroxide Ions in Aqueous Systems. *Chem. Rev.* **2016,** *116*, 7642-7672.
15. Ohshima, H., *Theory of Colloid and Interfacial Electric Phenomena*. Elsevier Academic Press: 2006.
16. Uematsu, Y.; Bonthuis, D. J.; Netz, R. R. Nanomolar Surface-Active Charged Impurities Account for the Zeta Potential of Hydrophobic Surfaces. *Langmuir* **2020,** *36*, 3645-3658.
17. Duignan, T. T.; Peng, M.; Nguyen, A. V.; Zhao, X. S.; Baer, M. D.; Mundy, C. J. Detecting the Undetectable: The Role of Trace Surfactant in the Jones-Ray Effect. *J. Chem. Phys.* **2018,** *149*, 194702.
18. Roger, K.; Cabane, B. Why Are Hydrophobic/Water Interfaces Negatively Charged? *Angew. Chem. Int. Ed.* **2012,** *51*, 5625-5628.
19. Yan, X.; Delgado, M.; Aubry, J.; Gribelin, O.; Stocco, A.; Boisson-Da Cruz, F.; Bernard, J.; Ganachaud, F. Central Role of Bicarbonate Anions in Charging Water/Hydrophobic Interfaces. *J. Phys. Chem. Lett.* **2018,** *9*, 96-103.
20. Carpenter, A. P.; Tran, E.; Altman, R. M.; Richmond, G. L. Formation and Surface-Stabilizing Contributions to Bare Nanoemulsions Created with Negligible Surface Charge. *Proc Natl Acad Sci USA* **2019,** *116*, 9214-9219.
21. Pullanchery, S.; Kulik, S.; Okur, H. I.; de Aguiar, H. B.; Roke, S. On the Stability and Necessary Electrophoretic Mobility of Bare Oil Nanodroplets in Water. *J. Chem. Phys.* **2020,** *152*, 241104.
22. Beattie, J. K.; Djerdjev, A. M. The Pristine Oil/Water Interface: Surfactant-Free Hydroxide-Charged Emulsions. *Angew. Chem. Int. Ed.* **2004,** *43*, 3568-3571.
23. Feng, J., et al. Single-Layer Mos2 Nanopores as Nanopower Generators. *Nature* **2016,** *536*, 197-200.
24. Hong, S.; Constans, C.; Surmani Martins, M. V.; Seow, Y. C.; Guevara Carrió, J. A.; Garaj, S. Scalable Graphene-Based Membranes for Ionic Sieving with Ultrahigh Charge Selectivity. *Nano Lett.* **2017,** *17*, 728-732.
25. Jabloński, J.; Janusz, W.; Szczypa, J. Adsorption Properties of the Stearic Acid-Octadecane Particles in Aqueous Solutions. *J. Dispersion Sci. Technol.* **1999,** *20*, 165-175.
26. Li, C.; Somasundaran, P. Reversal of Bubble Charge in Multivalent Inorganic Salt Solutions—Effect of Magnesium. *J. Colloid Interface Sci.* **1991,** *146*, 215-218.
27. Lützenkirchen, J.; Preočanin, T.; Kallay, N. A Macroscopic Water Structure Based Model for Describing Charging Phenomena at Inert Hydrophobic Surfaces in Aqueous Electrolyte Solutions. *Phys. Chem. Chem. Phys.* **2008,** *10*, 4946-4955.
28. Marinova, K. G.; Alargova, R. G.; Denkov, N. D.; Velev, O. D.; Petsev, D. N.; Ivanov, I. B.; Borwankar, R. P. Charging of Oil−Water Interfaces Due to Spontaneous Adsorption of Hydroxyl Ions. *Langmuir* **1996,** *12*, 2045-2051.
29. Secchi, E.; Niguès, A.; Jubin, L.; Siria, A.; Bocquet, L. Scaling Behavior for Ionic Transport and Its Fluctuations in Individual Carbon Nanotubes. *Phys. Rev. Lett.* **2016,** *116*, 154501.
30. Siria, A.; Poncharal, P.; Biance, A.-L.; Fulcrand, R.; Blase, X.; Purcell, S. T.; Bocquet, L. Giant Osmotic Energy Conversion Measured in a Single Transmembrane Boron Nitride Nanotube. *Nature* **2013,** *494*, 455-458.
31. Yang, C.; Dabros, T.; Li, D.; Czarnecki, J.; Masliyah, J. H. Measurement of the Zeta Potential of Gas Bubbles in Aqueous Solutions by Microelectrophoresis Method. *J. Colloid Interface Sci.* **2001,** *243*, 128-135.

32. Brown, M. G.; Walker, D. S.; Raymond, E. A.; Richmond, G. L. Vibrational Sum-Frequency Spectroscopy of Alkane/Water Interfaces: Experiment and Theoretical Simulation. *J. Phys. Chem. B* **2003,** *107*, 237-244.
33. Du, Q.; Freysz, E.; Shen, Y. R. Surface Vibrational Spectroscopic Studies of Hydrogen Bonding and Hydrophobicity. *Science* **1994,** *264*, 826.
34. Gragson, D. E.; Richmond, G. L. Comparisons of the Structure of Water at Neat Oil/Water and Air/Water Interfaces as Determined by Vibrational Sum Frequency Generation. *Langmuir* **1997,** *13*, 4804-4806.
35. Scatena, L. F.; Brown, M. G.; Richmond, G. L. Water at Hydrophobic Surfaces: Weak Hydrogen Bonding and Strong Orientation Effects. *Science* **2001,** *292*, 908.
36. Strazdaite, S.; Versluis, J.; Backus, E. H. G.; Bakker, H. J. Enhanced Ordering of Water at Hydrophobic Surfaces. *J. Chem. Phys* **2014,** *140*, 054711.
37. Yang, S.; Chen, M.; Su, Y.; Xu, J.; Wu, X.; Tian, C. Stabilization of Hydroxide Ions at the Interface of a Hydrophobic Monolayer on Water Via Reduced Proton Transfer. *Phys. Rev. Lett.* **2020,** *125*, 156803.
38. Pullanchery, S.; Kulik, S.; Rehl, B.; Hassanali, A.; Roke, S. Charge Transfer across C–H···O Hydrogen Bonds Stabilizes Oil Droplets in Water. *Science* **2021,** *374*, 1366-1370.
39. Jena, K. C.; Scheu, R.; Roke, S. Surface Impurities Are Not Responsible for the Charge on the Oil/Water Interface: A Comment. *Angew. Chem. Int. Ed.* **2012,** *51*, 12938-12940.
40. Okur, H. I.; Chen, Y.; Wilkins, D. M.; Roke, S. The Jones-Ray Effect Reinterpreted: Surface Tension Minima of Low Ionic Strength Electrolyte Solutions Are Caused by Electric Field Induced Water-Water Correlations. *Chem. Phys. Lett.* **2017,** *684*, 433-442.
41. Okur, H. I.; Drexler, C. I.; Tyrode, E.; Cremer, P. S.; Roke, S. The Jones–Ray Effect Is Not Caused by Surface-Active Impurities. *J. Phys. Chem. Lett.* **2018,** *9*, 6739-6743.
42. Marchioro, A.; Golbek, T. W.; Chatterley, A. S.; Weidner, T.; Roke, S. A Discrepancy of 107 in Experimental and Theoretical Density Detection Limits of Aerosol Particles by Surface Nonlinear Light Scattering. *Commun Chem* **2023,** *6*, 114.
43. Schönfeldová, T.; Okur, H. I.; Vezočnik, V.; Iacovache, I.; Cao, C.; Dal Peraro, M.; Maček, P.; Zuber, B.; Roke, S. Ultrasensitive Label-Free Detection of Protein–Membrane Interaction Exemplified by Toxin-Liposome Insertion. *J. Phys. Chem. Lett.* **2022,** *13*, 3197-3201.
44. Macias-Romero, C.; Nahalka, I.; Okur, H. I.; Roke, S. Optical Imaging of Surface Chemistry and Dynamics in Confinement. *Science* **2017,** *357*, 784-788.
45. Hermansson, K.; Bopp, P. A.; Spångberg, D.; Pejov, L.; Bakó, I.; Mitev, P. D. The Vibrating Hydroxide Ion in Water. *Chem. Phys. Lett.* **2011,** *514*, 1-15.
46. Buchanan, R. A.; Caspers, H. H.; Murphy, J. Lattice Vibration Spectra of Mg(Oh)2 and Ca(Oh)2. *Appl. Opt.* **1963,** *2*, 1147-1150.
47. Khatib, R.; Backus, E. H. G.; Bonn, M.; Perez-Haro, M.-J.; Gaigeot, M.-P.; Sulpizi, M. Water Orientation and Hydrogen-Bond Structure at the Fluorite/Water Interface. *Sci Rep* **2016,** *6*, 24287.
